# The Role and Function of HDL in Patients with Chronic Kidney Disease and the Risk of Cardiovascular Disease

**DOI:** 10.3390/ijms21020601

**Published:** 2020-01-17

**Authors:** Jacek Rysz, Anna Gluba-Brzózka, Magdalena Rysz-Górzyńska, Beata Franczyk

**Affiliations:** Department of Nephrology, Hypertension and Family Medicine, Chair of Nephrology and Hypertension, Medical University of Lodz, 90-549 Lodz, Polandbfranczyk_skora@wp.pl (B.F.)

**Keywords:** chronic kidney disease, high-density lipoprotein (HDL), dysfunctional, atherosclerosis, cardiovascular risk

## Abstract

Chronic kidney disease (CKD) is a worldwide health problem with steadily increasing occurrence. Significantly elevated cardiovascular morbidity and mortality have been observed in CKD. Cardiovascular diseases are the most important and frequent cause of death of CKD patients globally. The presence of CKD is related to disturbances in lipoprotein metabolism whose consequences are dyslipidemia and the accumulation of atherogenic particles. CKD not only fuels the reduction of high-density lipoprotein (HDL) cholesterol concentration, but also it modifies the composition of this lipoprotein. The key role of HDL is the participation in reverse cholesterol transport from peripheral tissues to the liver. Moreover, HDL prevents the oxidation of low-density lipoprotein (LDL) cholesterol by reactive oxygen species (ROS) and protects against the adverse effects of oxidized LDL (ox-LDL) on the endothelium. Numerous studies have demonstrated the ability of HDL to promote the production of nitric oxide (NO) by endothelial cells (ECs) and to exert antiapoptotic and anti-inflammatory effects. Increasing evidence suggests that in patients with chronic inflammatory disorders, HDLs may lose important antiatherosclerotic properties and become dysfunctional. So far, no therapeutic strategy to raise HDL, or alter the ratio of HDL subfractions, has been successful in slowing the progression of CKD or reducing cardiovascular disease in patients either with or without CKD.

## 1. Introduction

Chronic kidney disease (CKD) is a worldwide health problem with occurrence steadily increasing by approximately 6% annually, and significant differences in prevalence between populations [1,2]. According to the analysis performed for the Global Burden of Disease Study 2010, CKD-associated mortality almost doubled between 1990 and 2010; at the end of 2013, over three million people worldwide were undergoing renal replacement therapy (RRT), including two and a half million on hemodialysis or peritoneal dialysis, and nearly 700,000 who had received a kidney transplant [2,3]. Current estimations (2019) concerning the US population indicate that 15% of US adults (37 million people) have CKD [4]. This disease is more common in people aged 65 years or older (38%) in comparison to people aged 45–64 years (13%) or 18–44 years (7%) [4]. Analyses demonstrate that CKD is more prevalent in women (15%) than men (12%) and in non-Hispanic blacks (16%) than in non-Hispanic whites (13%) or non-Hispanic Asians (12%). The most worrying fact is that 9 out of 10 adults with CKD do not know they have it. In CKD, both the function and the structure of the kidneys are impaired [2]. Significantly elevated cardiovascular morbidity and mortality have been observed in the course of CKD. Cardiovascular diseases, which are the most important and frequent cause of death of CKD patients worldwide, are estimated to account for approximately 36% of deaths in this group of patients in Europe [5]. In patients with CKD, the cardiovascular mortality rate is 10 to 20 times higher compared to the general population, and in the end-stage renal disease (ESRD) population it is even 20–30 times higher [6,7]. The spectrum of cardiovascular diseases in the CKD population involves arterial vascular disease, such as atherosclerosis and arteriosclerosis, concentric left ventricular hypertrophy, heart failure, as well as non-atherosclerotic cardiovascular disease that becomes dominant at more advanced stages of CKD [8,9]. It is possible that casual factors underlying both CKD and cardiovascular disease (CVD) are similar, which creates the impression of the existence of association between these two diseases. The unravelling of the exact mechanisms and casual pathways linking CKD and CVD remains a great challenge. According to studies, increased cardiovascular risk in patients with CKD is multifactorial and involves both traditional (hypertension, diabetes, dyslipidemia) and kidney-specific risk factors, such as enhanced activity of the renin–angiotensin system (RAS) and sympathetic overactivity, endothelial dysfunction (related to the accumulation of asymmetric dimethylarginine, chronic inflammatory state, and oxidative stress), hyperphosphatemia as well as CKD-associated metabolic bone disorder (CKD-MBD) [8,10,11]. The carbamylation of low-density lipoprotein (LDL) is an example of the overlap between traditional and kidney-related risk factors. In addition, anemia, which is frequently observed in the course of CKD, stimulates the progression of left ventricular hypertrophy and increases cardiovascular morbidity [8]. In dialysis patients, the presence of abnormal bone metabolism, higher calcium–phosphorus levels, and diminished vitamin D levels are associated with vascular intimal and/or medial calcification. Moreover, increased cardiovascular risk is, in this group of patients, related to hemodynamic instability and hypotension. In turn, in patients who underwent kidney transplantation, the enhanced risk of cardiovascular morbidity and mortality is due to proteinuria, persistent inflammation (increased levels of serum C-reactive protein and IL-6), renal allograft dysfunction, moderate hyperhomocysteinemia, and anemia [8,12]. Disturbances in lipid profile are important factors associated with the development of cardiovascular diseases. The presence of chronic kidney disease is related to disturbances in lipoprotein metabolism which, in consequence, may lead to dyslipidemia and the accumulation of atherogenic particles [13]. Hypertriglyceridemia, higher levels of LDL cholesterol, lipoprotein(a) particles, and apolipoprotein B (Apo B)-containing lipoproteins as well as low high-density lipoprotein (HDL) levels are most frequent alterations observed in CKD patients. Chronic kidney disease not only fuels the reduction of HDL concentration, but it also modifies the composition of this lipoprotein, for example, by diminishing plasma levels of the main HDL components, namely apoA-I and apoA-II [14,15].

## 2. HDL Structure, Role, and Function

On the basis of their density, lipoprotein particles can be divided into chylomicrons, very low-density lipoproteins (VLDLs), low-density lipoproteins (LDLs), intermediate-density lipoproteins (IDLs) and high-density lipoproteins (HDLs) [16]. High-density lipoproteins are a heterogeneous group containing numerous subclasses differing in sizes, shapes, densities, protein compositions, and lipid diversity [17]. Their levels depend on body mass index (BMI), presence of diabetes, smoking and alcohol consumption habits, menopausal status (in women) as well as physical activity and dietary habits [18]. According to studies, serum concentration of HDL does not fluctuate significantly with age in male patients; however, in female patients, it tends to diminish, which is related to the lowering of estrogen activity [19].

### 2.1. Mature HDL Formation

Pre-β HDL is formed following the covalent binding of a lipid group to a lipid-free apoA-I. This particle becomes discoidal after accepting phospholipid and free cholesterol from hepatocytes and peripheral cells. Subsequent lipidation, cholesterol accumulation and esterification result in the formation of nascent spherical HDLs. Each spherical HDL particle contains approximately 50–130 phospholipids (such as phosphatidylcholines, sphingolipids, isoprenoids, acylglycerols), 30–90 cholesteryl ester molecules, 10–50 free cholesterol molecules, 10–20 triglyceride (TG) molecules, approximately 80 proteins (e.g., apoA-I and apoA-II) and enzymes [17,20,21,22]. The amount of cholesteryl esters and triglycerides in the hydrophobic core determines the size of HDL [17]. However, the type of transported proteins, lipids, and nucleic acids is responsible for many of the biological properties of HDL and its beneficial functions.

### 2.2. HDL Role and Functions

**Figure 1:**
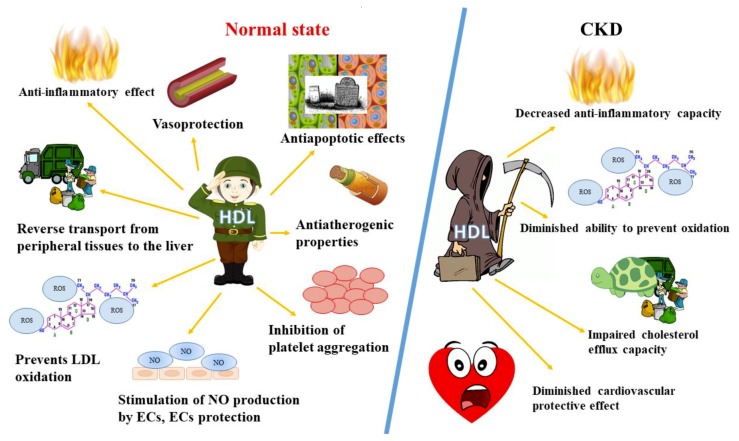
The summary of HDL functions in health and disease. CKD, chronic kidney disease; HDL, high-density lipoprotein cholesterol; LDL, low-density lipoprotein cholesterol; NO, nitric oxide; ECs, endothelial cells; ROS, reactive oxygen species.

It has been suggested that distinct HDL subspecies have differential biological functions. For example, large HDL seems to be less anti-inflammatory [23,24], whereas small HDL subclasses may be enriched in either cargo or other components of HDL that are responsible for its alternative functions [25,26]. The summary of HDL functions in health and disease is presented in Figure 1.

The key role of HDL is the participation in reverse transport of cholesterol from peripheral tissues to the liver. At the beginning of this process, cholesterol is extracted from peripheral tissues and this step is called cholesterol efflux. Then, the free cholesterol undergoes esterification by lecithin–cholesterol acyltransferase (LCAT) protein and it accumulates in the core of the HDL particle, which is associated with the transformation of lipid-poor discoid HDL particles to cholesterol-rich spherical HDL particles. Afterwards, HDL particles detach and are released into the bloodstream where cholesterol ester transfer proteins (CETPs) transfer part of the cholesterol content from HDL to IDLs and LDLs in exchange for their triglycerides [16]. In liver, HDL-C particles release lipid cargo and then lipid-poor HDL particles return to the circulation in order to repeat the cycle [15,16]. Experimental studies have demonstrated that under normal conditions HDL possesses antiatherogenic properties [15,27,28]. According to studies, HDL stimulates macrophage reverse cholesterol transport, which involves the extraction of excess cholesterol from lipid-laden macrophage foam cells present in the atherosclerotic plaque, the efflux of free cholesterol to mature HDL-C or extracellular lipid-poor apoA-I, and its transport to the liver for excretion in the bile [16,29]. Moreover, HDL prevents the oxidation of LDL by reactive oxygen species (ROS) and protects against the adverse effects of oxidized low-density lipoprotein (ox-LDL) on the endothelium, thus defending endothelial cell (EC) functions. The oxidation of LDL is a well-known process engaged in the development of atherosclerosis [17]. HDL absorbs oxidized lipids or oxidizing factors from cells preventing their attachment to LDL and it removes lipid hydroperoxides from LDL particles [30]. Small apoA-I-containing HDL particles efficiently accept lipid hydroperoxides and reduce them into inactive lipid hydroxides via the oxidation of methionine residues in apoA-I [17,31]. The ability of HDL to inhibit oxidation is also related to the selective elimination of HDL-bound lipid hydroperoxides and hydroxides by hepatocyte SR-BI [32]. Furthermore, HDL particles carry antioxidant enzymes, including serum paraoxonase/arylesterase 1 (PON1), lecithin–cholesterol acyltransferase (LCAT), platelet-activating factor acetylhydrolase (PAF-AH) and lipoprotein-associated phospholipase A2 (LpPLA2), which prevent lipid oxidation or degrade lipid hydroperoxides [17]. PON1 has been indicated to be a key regulator of HDL antiatherogenic properties [33,34]. Study results have indicated that higher activity of PON1 was associated with a lower incidence of major cardiovascular events in humans. HDL-associated LCAT exerts its antioxidative properties via the hydrolysis of oxidized acyl chains from phosphatidylcholine-based oxidized phospholipids and oxidized free fatty acids [34,35,36]. It has been demonstrated that HDL-associated platelet-activating factor acetylhydrolase (PAF-AH) hampers the production and limits the activity of mildly oxidized low-density lipoprotein since it facilitates the hydrolysis of active oxidized phospholipids to lysolipids, which in consequence results in the removal of biologically active lipids in these lipoproteins [37]. ApoA-I has been shown to be the main protein constituent of HDL which binds to and removes lipid hydroperoxides of LDL both in vitro and in vivo and reduces the amount of cholesteryl ester hydroperoxides and phosphatidylcholine hydroperoxides [31,38]. The resulting oxidized HDL was demonstrated to be removed by hepatocytes more rapidly and selectively than native HDL [31].

Numerous studies have demonstrated the ability of HDL to directly promote the production of nitric oxide (NO) by endothelial cells and to exert antiapoptotic and anti-inflammatory effects [29,39,40]. Uittenbogaard et al. [41] observed that HDL prevents oxLDL-mediated endothelial nitric oxide synthase (eNOS) displacement from caveolae, thereby helping to restore enzyme stimulation. NO-dependent atheroprotective effects of HDL enzyme, i.e., PON1 were implied by a study in which HDL isolated from PON1-deficient mice failed to stimulate NO production in mouse aortic endothelial cells [35,42]. Apart from the aforementioned apoA-I and PON-1, other HDL-associated apolipoproteins (e.g., ApoE) are also responsible for antioxidant effects of HDL particles [43,44]. For example, apoE2 was shown to stimulate endothelial NO release and apoE4, to exert proinflammatory effects, whereas apoA-IV showed antiatherosclerotic, anti-inflammatory, and antioxidant actions in vivo [45,46,47,48]. Small dense protein-rich HDL3 has been reported to counteract the inhibitory effects of ox-LDL on endothelium-dependent vasorelaxation in rabbit aortic rings, suggesting its potent vasorelaxing activity [49]. Moreover, Bisoendial et al. [50] suggested that endothelial function can be restored by increasing high-density lipoprotein levels in subjects with isolated decreased high-density lipoprotein concentration. The beneficial effects of HDL on endothelial cells is mainly mediated by phosphatidylinositol 3-kinase (PI3K)/Akt-dependent cyclin D1 activation [51].

Moreover, HDL actions are related to the differentiation of endothelial progenitor cells, improved cell survival and proliferation, cell migration, hampering of apoptosis and inflammation as well as limitation of adhesion molecule expression leading to greater vascular integrity [17,52]. Multiple antiapoptotic properties of HDL improve cell survival [17]. HDL helps to maintain endothelium barrier function, which seems to be a key feature of its antiatherosclerosis properties [17]. Another antiatherogenic function of HDL is related to its ability to hinder platelet activation, aggregation, and thrombus formation [17]. The inhibition of NF-kB activity by attenuating IκB kinase activity is the vital mechanism by which HDL suppresses endothelial cell and monocyte activation [17,53]. HDL has also been demonstrated to reduce adhesion molecule expression. In vitro studies demonstrated that HDL inhibited the expression of proinflammatory monocyte chemoattractant protein (MCP)-1 in endothelial cells [54,55].

Study results indicate that HDL levels are inversely associated with thrombus formation in humans [56]. Moreover, HDL was shown to alter platelet reactivity by binding specific receptors on platelet surfaces, which in consequence led to the activation of platelet signaling pathways. HDL also modulates the coagulation cascade at different levels, for example, via the inhibition of tissue factor and factors X, Va, and VIIIa. Zabczyk et al. [57] demonstrated the relationship between increased levels of HDL and enhanced fibrin clot permeability and shortened clot lysis time. In turn, Kaba et al. [58] and Asselbergs et al. [59] observed inverse association between both HDL level and HDL size and levels of PA inhibitor-I (PAI-I) (an inhibitor of tPA and uPA), which suggests that HDL particles may participate in the reduction of thrombotic risk via the stimulation of plasmin generation and thus fibrinolysis. On the other hand, ox-HDL, whose presence was shown in atherosclerotic plaques, promoted PAI-I expression in endothelial cells, which implies that ox-HDL decreases fibrinolysis and contributes to clot stability [60]. The beneficial effects of HDL, beyond its impact on vascular endothelium, involve suppression of myelopoiesis, limitation of monocyte recruitment, macrophage activation, and proliferation within atherosclerotic lesions [17].

## 3. HDL Disturbances

### 3.1. Chronic Kidney Disease

In CKD patients, especially in those undergoing dialysis, HDL levels are usually reduced [35] due to hampered maturation of HDL, which is related to diminished levels of apolipoproteins and LCATs [33] and its abnormal post-translational modifications [34]. Moreover, decreased concentration of HDL in CKD is associated with the decline in the metabolism of triglyceride-rich lipoproteins, the slower rate of apoA-I and A-II synthesis by the liver, and the enhanced activity of cholesteryl ester transfer protein (CETP) [61]. Modifications observed in HDL particles in pathological states impair cholesterol efflux [16,62], antioxidative functions, vasorelaxation, the ability to suppress TNFα-induced NF-κB activation and adhesion molecule expression, the capacity to inhibit TNFα-induced NADPH oxidase activity and superoxide production, the ability to metabolize hydroperoxides on erythrocyte membranes, the suppression of cytokine inhibition in activated inflammatory cells as well as antiapoptotic properties [16,17,63].

Increasing evidence suggests that, in patients with chronic inflammatory disorders, HDL may lose important antiatherosclerotic properties and become dysfunctional [17]. It turns out that HDL isolated from patients with inflammatory diseases, including diabetes, cardiovascular diseases, and chronic kidney disease, is less able to activate eNOS and its capacity to repair endothelium is also compromised [35]. The differences in HDL properties may be attributed to changes in the HDL-associated proteome and lipidome; such modifications involve alterations in the amount and type of proteins and lipids bound to the HDL particle as well as post-translational modifications. For the time being, at least several various mechanisms have been proposed to account for the impaired endothelial anti-inflammatory effects of HDL. In vitro, high-density cholesterol has been found to be susceptible to modifications by a variety of oxidants, including peroxyl and hydroxyl radicals, myeloperoxidase (MPO)-generated oxidants, metal ions, aldehydes, lipoxygenase, phospholipase, and to non-enzymatic glycation and homocysteinylation [64]. It has been suggested that diminished apoA-I levels in HDL in inflammatory states are associated with accelerated HDL catabolism and the substitution of apoA-I by serum amyloid A (SAA) [65,66]. Honda et al. [67] showed decreasing levels of HDL3 and ApoA-I in HDL3 subfraction along with the worsening of CKD severity; however, in their study, the level of HDL2 and ApoA-I in HDL2 subfraction was stable. The concentrations of ox-HDL were found to be similar across CKD stages [67]. Tolle et al. [68] demonstrated that HDL isolated from patients with chronic kidney disease was enriched in SAA and exhibited decreased anti-inflammatory capacity as assessed on the basis of its ability to inhibit monocyte chemoattractant protein-1 formation in vascular smooth muscle cells. Furthermore, the loss of PON1 diminishes the ability of HDL to prevent oxidation of its own lipids and proteins. As a consequence of PON1 shortage, HDL accumulates malonaldehydes, which limits NO production via increased phosphorylation of eNOS through LOX-1 receptor signaling [17,42].

Oxidative modifications of HDL are thought to contribute to the formation of dysfunctional proinflammatory HDL. Moreover, Ćwiklińska et al. [69] demonstrated that in CKD patients the positive effect of HDL on the efficiency of VLDL lipolysis was modified. They found that the efficacy of VLDL lipolysis in the presence of HDL was, in non-CKD patients, significantly higher than in those with CKD and it decreased with a reduction in estimated glomerular filtration rate (eGFR). It seems that HDL disturbances in CKD play a key important role in retarded VLDL catabolism and the development of hypertriglyceridemia. The increased levels of pre-β HDL subpopulation, which acts as LPL inhibitor, may explain the impact of HDL disturbances on VLDL lipolysis efficiency [69,70]. Additionally, modifications of VLDL and HDL composition and properties influencing their interactions may affect lipolysis; negative correlations were revealed between lipolysis efficiency and apoE and apoCs content in VLDL as well as a positive relationship between lipolysis and HDL–apoC-II and VLDL and HDL apoC-II/apoC-III ratios [69]. Moreover, Ćwiklińska et al. [69] observed that eGFR–MDRD (The Modification of Diet in Renal Disease Study equation) correlated with the adverse changes in both lipid and apolipoprotein composition of VLDL and HDL. For example, despite the reduction of HDL levels with worsening kidney function, the concentration of pre-β1 HDL was increasing with the progression of CKD. According to authors, decreased concentration and impaired HDL function along with an increase in triglycerides levels in CKD patients form a “vicious circle” in which these factors considerably influence one another, aggravating lipid disturbances and accelerating atherosclerosis development in this group of patients [69]. In chronic kidney disease, the cholesterol efflux capacity of HDL is also impaired, which is associated with structural changes of the HDL components [35]. Studies have demonstrated oxidative modification of apoA-I, mainly its methionine, tyrosine, or tryptophan residues via myeloperoxidase pathway, which resulted in considerably reduced ability of apoA-I to promote cholesterol efflux via ABCA1 pathway [35,71,72].

### 3.2. Nephrotic Syndrome

Impaired reverse cholesterol transport and subsequent enhanced foam cell formation, atherosclerosis, and glomerulosclerosis are also observed in the course of nephrotic syndrome. Heavy glomerular proteinuria and advanced CKD can stimulate profound changes in the structure and function of HDL [73]. Abnormalities in HDL particles in nephrotic syndrome are mainly related to the deficiency of lecithin–cholesterol acyltransferase (LCAT) resulting from urinary losses, hypoalbuminemia, increased plasma cholesterol ester transfer protein levels, and/or diminished expression levels of hepatic HDL docking receptor (SRB1) [73]. Moreover, deficiencies in ApoA-I, ApoA-II, paraoxonase-1, and glutathione peroxidase and increased levels of ACAT-1 are associated with HDL modifications. Enhanced oxidative and myeloperoxidase modifications to HDL cargo, including ApoA-I, ApoA-II, and SRB1, seem to be responsible for the impairment of HDL-mediated reverse cholesterol transport and HDL antioxidant and anti-inflammatory activity [73]. Moreover, in nephrotic syndrome, plasma cholesteryl ester transfer protein (CETP) has been demonstrated to be activated, which leads to the production of immature HDL [74]. Muls et al. [75] reported frequently impaired maturation of cholesterol ester-poor HDL3 to cholesterol ester-rich HDL2 in this population of patients. They suggested that the resulting impairment of reverse cholesterol transport could contribute to vascular complications. According to other studies, all modifications of HDL in patients with nephrotic syndrome contribute to endothelial dysfunction, accelerated atherosclerosis, and enhanced CVD risk.

### 3.3. Diabetic Nephropathy

In diabetic nephropathy (DN), HDL shows a higher level of glycation [76]. It was also less able to prompt cell migration both in vitro and in vivo in comparison to HDL from diabetes without nephropathy. The increased levels of glycated HDL could, in part, explain higher risk of cardiovascular disease in DN patients since such particles showed severely diminished capacity to stimulate endothelial cell migration. It seems that HDL capacity to stimulate cell migration is one of the mechanisms responsible for the protection of vascular vessels against damages [76].

### 3.4. Kidney Transplantation

In kidney transplant patients, the restoration of kidney function results in a significant increase in HDL (*p* < 0.0001); however, if the graft function is not maintained, HDL level reduction is observed (*p* < 0.01) [77]. In turn, Kopecky et al. [78] demonstrated that HDL from renal transplant recipients had strikingly altered molecular composition, mainly the enrichment in two proteins (SAA and SP-B), and it exerted impaired biological functions. Higher SAA content in HDL particles has been suggested to be associated with major cardiovascular risk [79], while plasma SP-B could be identified as a biomarker for chronic heart failure [80]. Kopecky et al. [78] found that, despite the fact that the distribution of most proteins seemed to be normal in transplant patients with functioning grafts, specific proteins were still enriched, which demonstrated a unique molecular composition of uremic HDL after transplantation. Moreover, they also observed compromised functional properties of HDL, including highly impaired cholesterol acceptor capacity. The ability of HDL to remove excess cholesterol from the periphery is believed to be of key importance in the prevention of atherosclerotic plaque formation [81,82]. HDL dysfunction turned out to be independent of graft function. It seems that alterations in HDL atheroprotective qualities pose one of the underlying reasons for the high cardiovascular morbidity and mortality in the transplant population [78]. These modifications of HDL particles may in part explain the diminished cardiovascular protective effect of HDLs found in the population of CKD patients; however, the presence of some of the HDL particle alterations and their effects should be confirmed in humans.

### 3.5. HDL and the Progression of Chronic Kidney Disease

Alterations in HDL cholesterol may also influence the progression of chronic kidney disease. Multivariate Cox regression analyses performed by Kawachi et al. [83] demonstrated a significant relationship between low HDL and ≥30% eGFR decline or ESRD (hazard ratio (HR) 4.80, *p* = 0.009), which was more apparent in the group of patients <70 years old (HR 4.96, *p* = 0.0165), especially in women (HR 13.86, *p*  =  0.0033). In a propensity score-matched cohort (CKD patients <70 years old), kidney survival rate was considerably diminished in the low HDL group in comparison to the high HDL group (*p* = 0.0364). Therefore, it seems that a low serum HDL level is a significant predictor of CKD progression, particularly in female patients with CKD under 70 years of age [83]. Two-sample Mendelian randomization analysis aimed at assessing the causal association between genetically determined lipid concentrations and kidney traits in CKD demonstrated a relationship between 17 mg/dL higher HDL cholesterol concentration and 0.8% higher eGFR (95% CI, 0.4–1.3%; *p* = 0.004) and lower risk for eGFR <60 mL/min/1.73 m^2^ (OR, 0.85; 95% CI, 0.77–0.93; *p* < 0.001) [84]. This observation supports the view that genetically higher HDL cholesterol concentration is causally associated with better kidney function.

### 3.6. Postmenopausal Women

According to numerous studies, in postmenopausal women a worse lipid profile is observed in comparison to premenopausal ones [85,86,87]. CKD women experience kidney dysfunction-mediated premature menopause. In the postmenopausal period, women are more prone to develop diseases associated with estrogen deficiency, including heart diseases, osteoporosis, and dyslipidemia [88,89,90] due to hormonal changes involving the decrease in estrogen level and increase in luteinizing hormone (LH) and follicle stimulating hormone (FSH) levels. These changes exert significant effect on plasma lipid and lipoprotein metabolism [89,91]. Estrogen cardioprotective effect are related to the maintenance of high levels of HDL and low levels of LDL-C and triacylglycerols [90]. Estrogen regulates HDL levels directly by influencing mRNA production for specific protein, for example, proteins involved in lipid metabolism including lipoprotein lipase (LPL) and hormone sensitive lipase (HSL) in adipose tissue, and by promoting hepatic expression of apoprotein gene as well as indirectly through the impact on adipose tissue by stimulating release of other hormones, namely, growth hormone (GH), catecholamine, and glucagon, which increases activity of HSL [90,92]. 17-Beta-estradiol, which is a main circulating form of estrogen, increases the rate of apoA-I and apoA-II synthesis leading to higher HDL concentration [90]. However, the issue concerning the level of HDL in postmenopausal women is controversial. Some studies have demonstrated significant lowering in the level of HDL in postmenopausal women, whereas other studies have noticed a substantial increase in level of HDL in this population. This discrepancy may be related to the differences in studied population, life intervention, and duration of menopause [90]. A cross-sectional study of premenopausal and postmenopausal women with metabolic syndrome (MS) and CKD failed to find significant changes in serum HDL cholesterol level between these two groups [93]. A large-scale cohort study found higher HDL levels in postmenopausal women [94]. In turn, Igweh et al. [95] demonstrated significant reduction in HDL in postmenopausal women when compared to premenopausal women. Additionally, a longitudinal study revealed a gradual increase in HDL from premenopausal period through menopausal transition to postmenopause. However, in this study, a slight decrease in HDL level was observed at late postmenopausal period [96]. Menopause-related hormonal alterations, mainly estradiol reduction, have been also suggested to promote the accumulation of risk factors resulting in chronic inflammation, which in consequence may impair the quality of HDL [97,98]. Zago et al. [99] revealed that HDL particles from postmenopausal women exhibited impaired antioxidant ability to limit LDL oxidation, implying that certain antiatherogenic properties of HDL are lost after the menopausal transition. The results of a collaborative EUROSTROKE study indicated that higher levels of HDL were associated with a considerably higher risk of nonfatal stroke and cerebral infarction in women aged ≥52 years [100].

## 4. Cardiovascular Risk Related to Modifications of HDL Particles in CKD Patients

In CKD patients and especially in ESRD patients requiring dialysis, the prevalence of coronary artery disease, sudden cardiac deaths, myocardial infarction, congestive heart failure, cerebrovascular disease, etc., is high [16,101,102,103,104]. Mortality from cardiovascular causes rises with the deterioration of renal function [16]. In ESRD patients, it is much higher than in the general population and in earlier CKD stages [16,103,105]. The results of some studies suggest that the modification of lipoprotein levels may not reduce the cardiovascular risk observed in the CKD population. For example, the reduction in LDL cholesterol after the treatment with atorvastatin in a 4D study (Die Deutsche Diabetes Dialyze Studie (German Diabetes and Dialysis Study)) had no statistically significant effect on the composite primary endpoint of cardiovascular death, nonfatal myocardial infarction, and stroke in patients with diabetes receiving hemodialysis [106]. Furthermore, the results of the AURORA trial demonstrated no relationship between rosuvastatin-related lowering of the LDL cholesterol level (by 43%) and the occurrence of composite primary endpoint of death from cardiovascular causes, nonfatal myocardial infarction, or nonfatal stroke in patients undergoing hemodialysis [107]. In turn, the Study of Heart and Renal Protection (SHARP) indicated a statistically significant 17% reduction in major cardiovascular events, but no significant reduction in mortality across a wide range of CKD stage [108], whereas the Prevention of Renal and Vascular End-stage Disease Intervention Trial (PREVEND IT) failed to demonstrate a significant reduction in cardiovascular events in microalbuminuric subjects treated with pravastatin [109]. Thus, it seems that the mechanism of cardiovascular risk in CKD reaches beyond lipid disorders and that the quality of lipoproteins may be more important than their levels.

Numerous epidemiological studies have demonstrated an inverse relationship between HDL levels and the risk of CVD [17,110]. However, more recent results suggest that persons with high HDL levels are not protected from cardiovascular diseases and that raising HDL levels through appropriate treatment (e.g., the administration of niacin and CETP inhibitors) does not reduce the risk of cardiovascular events [111]. Moreover, the data concerning the association of HDL with cardiovascular mortality in CKD and ESRD patients is inconsistent. Shoji et al. [112] revealed that the assessment of HDL enabled the prediction of incident myocardial infarction in Japanese patients on hemodialysis with no history of CVD. In contrast, several European studies failed to demonstrate any relationship between HDL and all-cause or cardiovascular mortality [113,114]. In a large study of >33,000 hemodialysis patients, U-shaped association between HDL concentrations of <30 mg/dL and >60 mg/dL and increased risk for total and CVD mortality was observed [115,116]. The results of the Investigation of Lipid Level Management to Understand its Impact in Atherosclerotic Events (ILLUMINATE) trial assessing the impact of torcetrapib (CETP inhibitor) on clinical outcome indicated that despite the fact that the treatment significantly increased HDL levels, the risk of mortality and morbidity in patients at high risk for coronary events remained unchanged [117]. Moreover, reduced serum HDL concentrations in ESRD has not been linked with altered survival or the occurrence of cardiac events [118,119,120].

As it has been mentioned before, in some pathological states (such as CVD, type 2 diabetes (T2D), and CKD), HDL loses its beneficial properties and becomes dysfunctional. In the course of CKD, HDL particles not only lose their atheroprotective properties but are also converted into deleterious molecules. It seems that chronic kidney disease may promote an inflammatory microenvironment that could stimulate atherogenic transformation of the HDL proteome and impair HDL function. HDL in CKD has been shown to have decreased ability to promote cholesterol efflux from macrophages and impaired antioxidative, anti-inflammatory and vasoprotective properties [69,121,122]. According to studies, in CKD patients, the distribution of HDL subfractions is altered. High levels of small HDL particles were suggested to correlate with lower probability to develop atherosclerosis, whereas large HDL particles were shown to be proatherogenic [16,123]. Therefore, low levels of large HDL particles and high levels of small HDL particles seem to protect against the incidence of cardiovascular events [16,124]. Kuchta et al. [125] demonstrated that, in CKD patients with stages 3a-4, the concentration of preβ1-HDL was increased. Elevated concentrations of preβ1-HDL have been also observed in patients with coronary artery disease, which suggests the relationship between higher levels of preβ1-HDL and the risk of CVD [126,127]. Kuchta et al. [125] reported a substantial increase of preβ1-HDL in patients with 3a–4 stages of CKD, which was probably associated with a compromised LCAT-dependent conversion of preβ1-HDL into α-migrating HDL. Other studies have confirmed that CKD decreased both levels and activity of circulating LCAT, and in advanced stages of the disease, it downregulated hepatic LCAT gene expression [128,129]. The results of other studies indicate a relationship between recent hemodialysis initiation and higher abundance of eight HDL-associated proteins, which were recognized as markers or mediators of inflammatory, atherosclerotic, and lipid metabolism pathways [130]. The remodeling of HDL in ESRD involves the acquisition of new proteins, such as SAA or SP-B [78,131]. In turn, the enrichment of HDL with APOE can interfere with HDL metabolism and initiate atherogenesis [73,118,130]. Chronic kidney disease severely alters enzyme activities involved in HDL metabolism; it affects particle maturation and remodeling, which in consequence alters HDL composition and function [132,133,134]. Compositional alterations also include, apart from the aforementioned enrichment of HDL with proinflammatory acute-phase proteins, considerable reduction of HDL-paraoxonase content and activity [78,132,135]. Holzer et al. [121] reported lower relative levels of APOA1 and APOA2 and higher levels of SAA1, albumin, and APOC3 in hemodialysis patients in comparison to healthy controls. This observation was confirmed by other studies [135,136]. Some studies suggest the relationship between chronic hemodialysis and elevated HDL concentrations of APOC2, lipoprotein-associated phospholipase A2, α1-microglobulin/bikunin precursor, surfactant protein, α1-antitrypsin, APOA4, α1-acid glycoprotein 2, RBP4, HPR, and transthyretin, which are mostly acute-phase reactants and key mediators of immune response and thrombosis pathways [121,135,136]. In African Americans, APOL1 alleles G1 and G2 have been hypothesized to contribute to the elevated CVD risk [137]. This suggestion might be biologically plausible, as APOL1 is a major apo component of HDL3 particles, which plays a key role in cholesterol transport and the limitation of low-density lipoprotein (LDL) oxidation [138]. These two APOL1 alleles are also associated with a higher incidence of CKD (including focal segmental glomerulosclerosis, collapsing glomerulopathies, and arterionephrosclerosis) and a more progressive course of the disease in the African American population [2,139,140]. The Jackson Heart Study demonstrated a two-fold increase in the risk of cardiovascular events in carriers of high-risk genotype (G1/G1, G2/G2, or G1/G2). Ito et al. [137] revealed that the relationship between APOL1 alleles and coronary artery disease risk seemed to be independent of renal disease. Moreover, in a study using the population from the Women’s Health Initiative, no association was found between the high-risk genotype and cardiovascular event precursors, including left ventricular hypertrophy [137]. Ryu et al. [141] showed that APOL1-G1 and -G2 genetic variants were associated with higher intracellular cholesterol content, ABCA1 and ABCG1 suppression, and, in consequence, with reduced reverse cholesterol transport. The results of their study suggest that impaired cholesterol efflux capacity observed in APOL1 renal risk variant macrophages may stimulate, in vivo, the formation of foam cells. Moreover, the presence of APOL1 renal risk variants have been demonstrated to be related to decreased plasma concentrations of medium-sized HDL; this may mean that APOL1 variants limit the production of medium-sized HDL [142]. Due to the fact that medium and small HDL particles exert protective activity against cardiovascular disease, it seems that APOL1 variants may promote the increase in CVD risk. A better understanding of APOL1 biology will enable the unravelling of the mechanism through which APOL1 alleles promote the development of CVD and the answering of the question whether they are indeed involved in the increased CVD risk [137].

Kollerits et al. [143] demonstrated that increased risk for all-cause mortality and sudden cardiac death in patients with CKD on hemodialysis was related to low apoA-IV levels. In dialysis patients, higher levels of HDL-associated SAA have been shown to result in impaired HDL cholesterol efflux capability [121] and to be associated with cardiovascular mortality [130,144]. Post hoc analysis of the 4D Study revealed an independent association between HDL-associated proteins, namely SAA and surfactant protein B (SP-B) and cardiac events and overall mortality [144]. Untersteller et al. [132] found that higher SAA levels and lower paraoxonase activity were predictors of adverse outcome. However, following the adjustment for traditional cardiovascular and renal risk factors, only the relationship of SAA with the occurrence of the primary endpoint remained significant.

Two meta-analyses found that asymmetric dimethylarginine (ADMA), but not symmetric dimethylarginine (SDMA), was strongly associated with CVD outcomes in patients with kidney diseases [145,146]. Moreover, it has been demonstrated that advanced oxidation protein products (AOPPs), which are markers of oxidative stress carried by oxidized plasma proteins such as oxidized albumin, accumulate in renal disease, bind with high affinity to SR-B1, block the binding of HDL to SR-B1, and therefore limit cholesterol ester uptake [14,147]. Kalantar-Zadeh et al. [148], in a study of nearly 200 hemodialyzed CKD patients, found that the HDL-inflammatory index correlated with poor outcome. In a study of postmenopausal women with an HDL level >60 mg/dL and a very low CVD risk, higher levels of HDL were shown to be independently associated with a higher prevalence of carotid atherosclerosis [149]. The result of a systemic review showed a higher risk of CVD, CVD mortality, and all-cause mortality in women who experienced early menopause [150]. Finally, a longitudinal Study of Women’s Health Across the Nation (SWAN) [98] provided the evidence that supports the hypothesis that a higher level of HDL in middle-aged and older women is not always protective. Despite most of the aforementioned studies concerning menopausal women did not focus on the CKD population, we believe that the findings presented in that study are also true for women with chronic kidney disease.

## 5. Conclusions

Under normal conditions, the range of antiatherogenic effects of HDL includes its participation in reverse cholesterol transport, and antioxidative, anti-inflammatory, and antiapoptotic activity [35]. However, growing evidence indicates that HDL particles are highly heterogeneous and, under pathological conditions, including CKD, HDL properties may be altered, resulting in increased cardiovascular risk. Therefore, further studies are required to explore the particular mechanisms responsible for the loss of HDL’s beneficial properties. So far, no therapeutic strategy to raise HDL, or alter the ratio of HDL subfractions, has been successful in slowing the progression of CKD or reducing cardiovascular disease in patients either with or without CKD.
